# Spectroscopic, Theoretical and Antioxidant Study of 3d-Transition Metals (Co(II), Ni(II), Cu(II), Zn(II)) Complexes with Cichoric Acid

**DOI:** 10.3390/ma13143102

**Published:** 2020-07-11

**Authors:** Grzegorz Świderski, Agata Jabłońska-Trypuć, Monika Kalinowska, Renata Świsłocka, Danuta Karpowicz, Marta Magnuszewska, Włodzimierz Lewandowski

**Affiliations:** 1 Department of Chemistry, Biology and Biotechnology, Bialystok University of Technology, Wiejska 45E Street, 15-351 Bialystok, Poland; a.jablonska@pb.edu.pl (A.J.-T.); m.kalinowska@pb.edu.pl (M.K.); r.swislocka@pb.edu.pl (R.Ś.); d.karpowicz@pb.edu.pl (D.K.); marta.mag@poczta.onet.pl (M.M.); 2 Department of Food Analysis, Institute of Agricultural and Food Biotechnology, Rakowiecka 36 Street, 02-532 Warsaw, Poland

**Keywords:** cichoric acid, caffeic acid, antioxidant properties, metal complexes

## Abstract

Cichoric acid (CA) is a derivative of both caffeic acid and tartaric acid. It was isolated for the first time from *Cichorium intybus* L. (chicory) but it also occurs in significant amounts in *Echinacea*, particularly *E. purpurea*, dandelion leaves, basil, lemon balm and in aquatic plants, including algae and sea grasses. It has a wide spectrum of biological properties, including antioxidant, antiviral, anti-inflammatory and other. The work yielded cichoric acid complexes with selected transition metals, i.e., copper(II), nickel(II), zinc(II) and cobalt(II). In this work the dependency between the molecular structure and biological activity was discussed. The molecular structure was studied by means of infrared spectroscopy (Fourier transform infrared (FT-IR) Raman (FT-Raman)), electronic absorption spectroscopy (ultraviolet–visible (UV/VIS)) and theoretical calculations (density functional theory (DFT), Hartree–Fock (HF)). Understanding the mechanism of the effect of metals on the electronic system of ligands with biological importance will facilitate in the future the search for new, effective and natural antioxidants. The composition of the studied complexes in aqueous solutions was determined at a constant pH by the Job’s method. Antioxidative properties of the tested compounds were determined using the ferric-reducing antioxidant power (FRAP), DPPH (2,2-diphenyl-1-picryl-hydrazyl-hydrate free radical method), cupric-reducing antioxidant capacity (CUPRAC) and Superoxide Dismutase Activity Assay (SOD).

## 1. Introduction

**Scheme 1:**
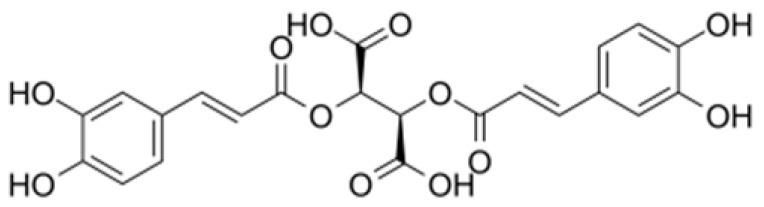
Structural formula of cichoric acid.

Natural antioxidants play a large role in the prevention of many diseases. They remove reactive oxygen species and in such a way protect cells and reduce oxidative damage. A properly selected diet, rich in ingredients with a healthy effect, including natural antioxidant compounds, has a huge impact on the repair processes of the organism, and can prevent many diseases, among others cancer. Natural substances with high antioxidant activity are among others phenolic acids such as caffeic, ferulic, gallic, ellagic and many others. Phenolic acids in plants usually occur in bound form as components of lignins, tannins, as esters or glycosides. They can also be present in free form or as depsides (e.g., chlorogenic acid, rosemarinic acid, cichoric acid, cynarin). Among the phenolic acids, three basic groups of compounds can be distinguished: hydroxycinnamic, hydroxybenzoic and phenylacetic acids. Derivatives of the first two acids are secondary metabolites which are often found in foods of plant origin [1]. Hydroxycinnamic acids commonly found in plants and dietary products are caffeic, chlorogenic (an ester of caffeic and quinic acids), o-, m- and p-coumaric, ferulic and synapinic acids. Caffeic acid is characterized by high antioxidant potential. Caffeic acid occurs, among others, in coffee, apples, potatoes, cabbage, wine and other products. There are several derivatives of caffeic acid with equally high antioxidant potential: rosmarinic acid (an ester of 3,4-dihydroxyphenylacetic and caffeic acids), chlorogenic acid, cynarin (dicaffeoylquinic acid) and cichoric acid. Cichoric acid (CA) (Scheme 1) is a derivative of both caffeic acid and tartaric acid. It was isolated for the first time from *Cichorium intybus L.* (chicory) but it also occurs in significant amounts in *Echinacea*, particularly *E. purpurea L.*, dandelion leaves, basil, lemon balm and in aquatic plants, including algae and sea grasses [2,3]. Extracts of *Echinacea purpurea L.* (containing, among others, cichoric acid, caftaric acid and other antioxidant compounds) are widely used as anti-inflammatory substances to treat colds, coughs, bronchitis and upper respiratory tract diseases. Cichoric acid is an important bioactive phenolic compound with high antioxidant potential [4]. It has a catechol ring, which largely explains its high antioxidant activity [5]. CA has also an antivirus property. It was found that cichoric acid and its analogues show activity against human immunodeficiency virus (HIV) due to its participation in the inhibition of HIV integrase, which may inhibit the development of the virus [6,7]. A diet rich in cichoric acid effectively reduces body weight and has a hypoglycaemic effect [8]. Studies shown that cichoric acid reduces fatty acid concentrations in serum and liver, significantly reduces liver steatosis, prevents inflammatory reactions in the liver of obese mice by regulating the release of inflammatory cytokines and inactivating JNK (stress-activated kinases) [9]. Cichoric acid promotes insulin secretion and regulates blood glucose levels by inhibiting pancreatic apoptosis and tissue damage in diabetic mice, further regulates mitochondrial biogenesis, improves glycogen synthesis and suppresses inflammation by activating the antioxidant response [10]. Cichoric acid exhibits anticancer properties. It was shown that cichoric acid decreases cell viability and induces apoptosis in gastric cancer cells. Moreover, it prevents tumor growth in an established xenograft gastric cancer model [11]. Cichoric acid strongly inhibits the growth of colorectal cancer cells [12].

In addition, studies have shown that transition metal complexes with phenolic acids or flavonoids are characterized by higher bioavailability than pure ligands. For example, due to its low solubility, quercetin has poor bioavailability in vivo, but transition metal (such as Cu(II), Fe(II), Mn(II)) complexes with quercetin show increased bioavailability [13]. Low water solubility and poor bioavailability of cichoric acid limit its clinical use [14]. Therefore the absorption of cichoric acid can be improved by metal chelation. Studies have shown that extracts from the *Echinacea* plant possess antimicrobial capacity. This plant contains a number of phenolic compounds and it is a rich source of cichoric acid. Many studies revealed that metal complexes with phenolic acids often show an increase in their antimicrobial activity [15,16]. Cu(II), Zn(II), Ni(II) and Co(II) complexes with cichoric acid have higher antimicrobial potential than ligand alone, which may also result from its greater absorption. Chelation of metal ions such as iron (element essential for the body and consumed with food) by cichoric acid and its derivatives, or by other biologically active components of plants (including phenolic compounds) can affect both the body’s absorption of metals and also may increase antimicrobial activity ligands.

In the framework of this study the physicochemical and biological properties of cichoric acid, its sodium(I) salt, and complexes with copper(II), cobalt(II), nickel(II) and zinc(II) were investigated. Many reports have demonstrated that metal chelates can be more efficient free radical scavengers than the phenolic compounds alone [17,18,19], e.g., the complexes of Cu(II), Fe(II) and Fe(III) with kaempferol, quercetin, rutin and epicatechin [20,21] and the compounds of curcumin with Cu(II) and Zn(II) [22]. The formation of a complex of metal ion with a phenolic compound not only changes the physicochemical properties of the ligand (including increase in the stability or changes in the lipophilicity), but also its biological activity, e.g., antioxidant, antimicrobial and many others [23,24]. Our previous papers concerned the physicochemical and antimicrobial properties of metal complexes with phenolic acids naturally occurring in foods, e.g., ferulic, caffeic and p-coumaric acids with alkali metals, manganese(II), copper(II) and cadmium(II) [25,26].

This paper is a part of a broad research topic the aim of which is to: (1) study the correlation between the molecular structure and biological activity (antimicrobial, antioxidant and cytostatic) of selected phenolic compounds and their metal complexes, (2) search for new effective natural antioxidants. In our studies we discuss how metal complexation affects the molecular structure and electronic charge density of molecule and whether these changes influence on its biological activity. The fragmentary literature and our own data shown that the complexation of metals by ligands improves the antioxidant capacity of these ligands. The antioxidant activity often increases even by an order of magnitude or higher after metal complexation. Therefore, the important question arises: what are the mechanisms of the observed dependencies and which properties of metal determine the improvement or decrease of the biological activity of the complex compared to the ligand. Among others, the effect of metal ions on radicals or anti- and pro-oxidant properties of ligands depends on: redox potential, ability to generate the Fenton’s reaction, concentration of reagents. The next question is: what other parameters of metals can affect the antioxidant capacity of ligands when the are bound to metal ions. Is the electronegativity, ionic radius, effective charge of metal ion or ionic potential decisive? Moreover, the physico-chemical properties of ligands also affect their antioxidant properties. Our initial studies revealed that beside the number and position of the hydroxyl group in the phenolic ring [27], other factors are very important as well, i.e., the length of the conjugated double bond system, the degree of the electronic charge delocalization, aromatic properties of phenolic ligands measured by the use of aromaticity indices and the donor-acceptor properties described by the energy of HOMO (Highest Occupied Molecular Orbital) and LUMO (Lowest Unoccupied Molecular Orbital) orbitals. The effect of these metal and ligand properties should be carefully analysed in the context of the anti- and pro-oxidant properties of metal complexes. Of course, this issue is not easy. Simple dependencies are not expected because various factors may overlap the observed final effects.

## 2. Materials and Methods

### 2.1. Sample Preparation

#### 2.1.1. Synthesis of Cichoric acid Sodium Salt

0.02 mmol of cichoric acid was weighed and dissolved in 0.40 mL NaOH solution at a concentration of 0.1 M. 2.6 mL of deionized water was added to the mixture. The solution was stirred in a water bath at 50 °C by 1 h. The molar ratio of ligand:metal was 1:2. The solution was allowed to evaporate slowly and the precipitate was air-dried at 30 °C.

#### 2.1.2. Synthesis of Cu(II), Co(II), Zn(II) and Ni(II) Complexes with Cichoric Acid

The weighed mass of cichoric acid was added to with appropriate volume of NaOH (0.1 M) in a stoichiometric molar ratio 1:1 and stirred at 50 °C by 1 h. Next, the aqueous solution of transition metal chloride was added to the mixture in order to obtain the molar ratio of ligand:transition metal cation 1:2. Then it was left for 2 h at room temperature in a shaker. After several days, precipitates occurred. They were filtered from the solution and washed with deionized water. The precipitates were air-dried at 30 °C. The obtained yield of the synthesis was ~70–85%. Complexes with a ligand:metal ratio (1:2) were obtained.

The chemicals: CuCl_2_·2H_2_O, ZnCl_2_, CoCl_2_·6H_2_O, NiCl_2_·6H_2_O, NaOH, cichoric acid, tris(hydroksymetylo)aminometan, TRIS–HCl, methanol, DPPH, ABTS, KBr from the Sigma-Aldrich (St. Louis, MO, USA).

#### 2.1.3. Elemental Analysis Results

C_22_O_12_H_16_Na_2_·2H_2_O: H_exp_%/H_calc_%: (3.53/3.45), C_exp_%/C_calc_%: (46.82/46.97); C_22_O_12_H_16_Zn_2_Cl_2_·4H_2_O: (3.30/3.24), (35.59/35.42); C_22_O_12_H_16_Ni_2_Cl_2_·4H_2_O: (3.42/3.32), (36.21/36.06); C_22_O_12_H_16_Cu_2_Cl_2_·2H_2_O: (2.91/2.85), (37.60/37.41); C_22_O_12_H_16_Co_2_Cl_2_·4H_2_O: (3.35/3.30), (36.18/36.04).

### 2.2. Measurements

#### 2.2.1. Infrared (IR) and Raman Study

An infrared (IR) and Raman study of the samples in solid state was conducted: the Fourier transform infrared (FT-IR) spectra for the samples in KBr matrix pellets were recorded with the use of Alfa (Bruker, Billerica, MA, USA) spectrometer in the range of 400–4000 cm^−1^. FT-Raman spectra were recorded with a MultiRam (Bruker) spectrometer in the same range of cm^−1^.

#### 2.2.2. Ultraviolet (UV) Study

The spectra of studied compounds at the concentration of 5 × 10^−5^ mol/L were recorded in the range of 210–400 nm using a HACH apparatus 5000 DR spectrophotometer.

#### 2.2.3. Calculations

The optimized structures were calculated using quantum–mechanical methods: the density functional (DFT) hybrid method B3LYP with non-local correlation provided by Lee–Young–Parr expression and HF (Hartree–Fock) was used. All calculations were carried out with functional base 6-311+G(d,p) (the base included H-Kr atoms). Calculations were performed using the Gaussian 09 (Frisch et al., 2009) package [28]. The experimental FT-IR spectra were interpreted in terms of DFT and HF calculations. Theoretical wavenumbers were scaled according to the formula: ν_scaled_ = 0.98·ν_calculated_ for B3LYP/6-311++G(d,p) level and ν_scaled_ = 0.89·ν_calculated_ for HF/6-311++G(d,p) level of calculations. The energy of HOMO or LUMO orbitals and selected electronic parameters were calculated. The calculated complexes are cations (with a 2+ charge) in which the presence of a counterion (Cl^−^ chloride ion) was not taken into account. The presence of water in the structures was not included in the calculations. Full structures of the complexes (neutral structures) have not been theoretically modeled. For hydrated structures and containing chloride counterion, the calculated structures had imaginary frequencies (transient structures). The analysis of the results was based on optimized complex cations, taking into account only the effect of metal on the structure and properties, excluding the contribution of counterion and water.

#### 2.2.4. Antioxidant Properties

DPPH (2,2-diphenyl-1-picryl-hydrazyl-hydrate free radical method): antiradical activity was measured according to the DPPH assay described in [29]. The final concentration of studied compounds was in the range of 2–20 μM. The control sample consisted of 2 mL of DPPH solution and 1 mL of methanol. The absorbance of the mixture was measured at 516 nm against methanol as the blank using a NANOCOLOR VIS spectrophotometer (Düren, Germany). The antiradical activity of CA and its complexes was calculated according to the equation:

$$\;I\%=\frac{A_{control}^{516}-A_{sample}^{516}}{A_{control}^{516}}\cdot 100\%$$

where: I%—percent of inhibition of DPPH radical,$\;A_{conrol}^{516}$—absorbance of the control sample, $A_{sample}^{516}$—absorbance of the tested sample.

FRAP: ferric-reducing antioxidant activity was determined by the use of a ferric-reducing antioxidant power (FRAP) assay according to the procedure described in [30]. The FRAP reagent (2.5 mL) was mixed with tested substance (50 µL; final concentrations C = 6.0 µM). The absorbance was measured at 594 nm against blank using a NANOCOLOR VIS spectrophotometer. Antioxidant activity was expressed as Fe^2+^ equivalents [µM] using the calibration curve prepared over the range of 100–20 µM concentration of FeSO_4_.

CUPRAC: a cupric-reducing antioxidant activity (CUPRAC) assay was conducted according to [31]. 3 mL of this CUPRAC solution, 0.5 mL cichoric acid or its metal complex (final concentrations C = 6.0 µM) and 0.6 mL deionised water were mixed. After 1 h the absorbance was measured at 450 nm using a Nanocolor Vis spectrophotometer. Antioxidant activity was expressed as trolox equivalents [mM] by using the calibration curve obtained for trolox in the range of concentration 0.001–0.2 µM.

SOD activity: the SOD-mimic activities were determined by the indirect method based on the competitive reaction of the cichoric acid complexes and XTT dye [2,3-bis(2-methoxy-4-nitro-5-sulfophenyl)-2tetrazolium-5-carboxanilide natrium salt] with a saturated DMSO (dimethyl sulfoxide) solution of potassium superoxide (KO_2_) [32]. The 100 µL solution of tested substance was mixed with 2 mL of phosphoric buffer (pH = 7.4; C = 0.01 M). Then 50 µL XTT dye in DMSO, 100 µL of saturated KO_2_ in DMSO were added. The final concentration of tested compounds was in the range of 0.1–20 μmol/L. The samples were incubated 30 min in room temperature. The control sample was a solution containing the same substances and 100 µL DMSO instead of tested compound. The absorbance was measured at 480 nm. The percentage of inhibition was calculated according to the formula:

$$\;\mathrm{Inhibition}\%=\frac{A_{0\;}-{\;A}_{i}}{A_{0}}\cdot 100\%=\left(1-\frac{A_{i}}{A_{0}}\right)\cdot 100\%$$

A_0_—absorbance of the control sample, A_i_—absorbance of the tested sample.

## 3. Results and Discussion

### 3.1. IR and Raman Spectra

**Figure 1:**
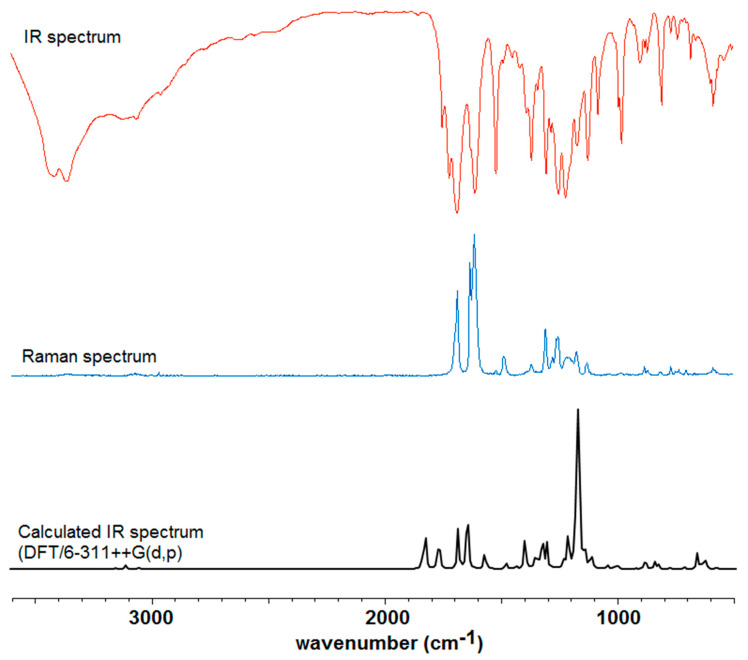
Experimental infrared (IR), Raman and calculated IR spectra of cichoric acid.

**Figure 2:**
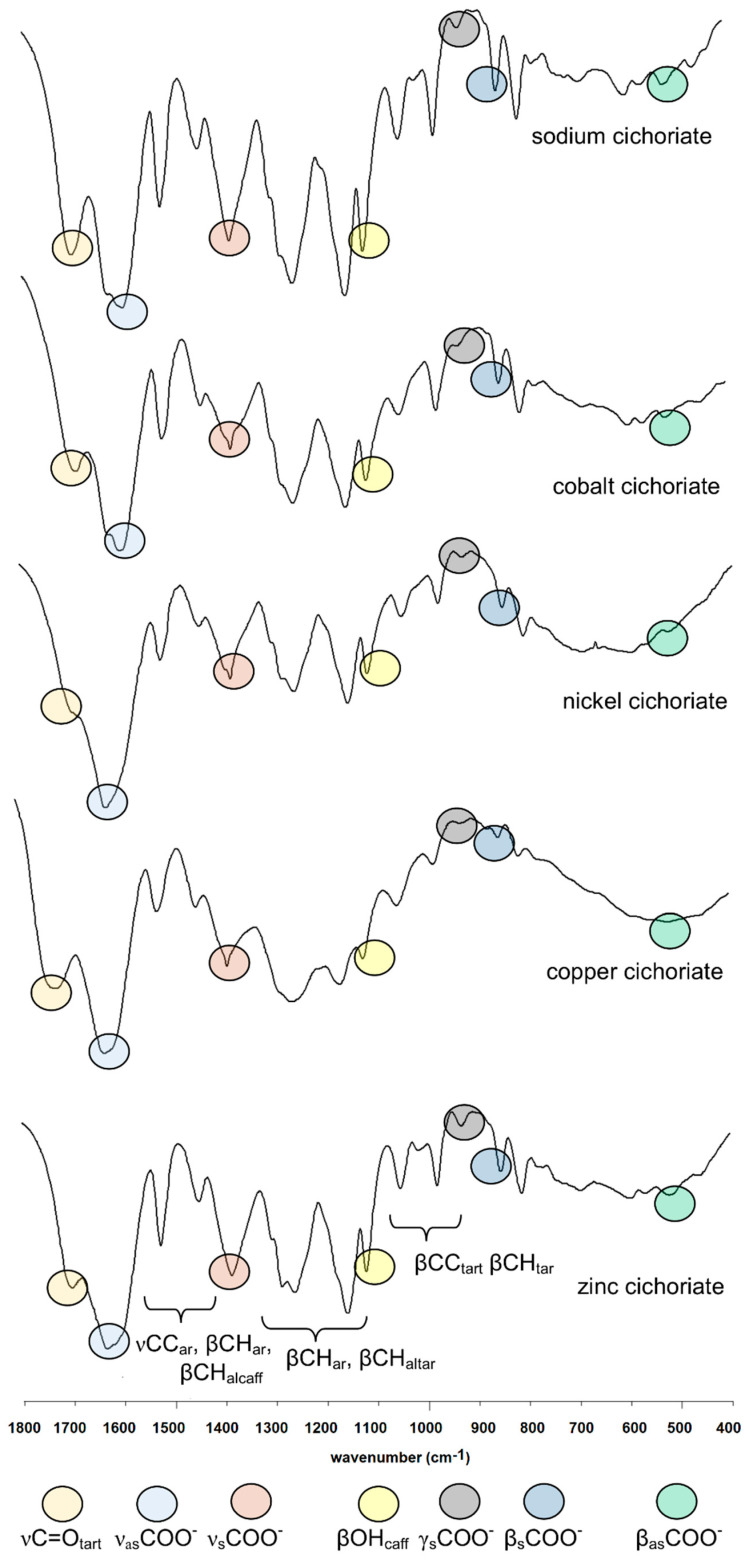
IR spectra of sodium salt and Co(II), Ni(II), Cu(II) and Zn(II) complexes of cichoric acid (400–1800 cm^−1^).

The calculated IR spectra of cichoric acid and its complexes were used in order to properly assign the experimentally obtained spectra for these compounds. High consistency was obtained between the experimentally and theoretically obtained wavenumbers (correlation coefficient R > 0.98). The Figure 1 shows the IR, Raman and theoretical spectra of cichoric acid. Figure 2 shows the IR spectra of sodium salt and complexes with copper(II), zinc(II), nickel(II) and cobalt(II). Table 1 presents the experimental and theoretical spectral data of the studied complexes. Interpretation and assignment of these bands is quite difficult because a series of bands overlaps. Whereas in the experimental spectra a much smaller number of bands occurred than in calculated spectra.

In the IR and Raman spectra of cichoric acid there were many bands that originate from vibrations of the caffeic and tartaric acid moieties. In the IR spectrum of the acid the bands at 1746 and 1716 cm^−1^ were interpreted as vibrations of the carboxyl group of the tartaric acid moiety. In the IR spectra of metal complexes these bands disappeared what indicates that the metal is bond to the cichoric acid through the carboxylate group of the tartaric acid. In the spectra of the complexes characteristic wide bands originating from vibrations of the carboxylate anion appeared. These are, among others, the bands derived from the stretching vibrations of the symmetric carboxylate anion ν_sym_COO^−^ (1385 cm^−1^–1384 cm^−1^) and stretching asymmetric vibrations ν_as_COO^−^: (1630 cm^−1^–1605 cm^−1^) (Table 1, Figure 1). Moreover in the spectra of complexes bands assigned to the symmetric bending in-plane (β_s_COO^−^) occurred (868 cm^−1^–851 cm^−1^) and asymmetric bending β_as_COO^−^ (521 cm^−1^–520 cm^−1^) (Table 1).

In the IR and Raman spectra of CA and its complexes and sodium salt, a series of bands derived from vibrations of the aromatic ring (caffeic acid moiety) were assigned. Many bands present in the spectra of cichoric acid changed their intensity and location after metal complexation. It is caused by the influence of metal ions on the electronic charge density of ligand. These were bands assigned to the stretching vibrations of the carbon–carbon bond (νC–C) located approximately at 1606, 1517, 1300 cm^−1^ (in the spectrum of CA), bands derived from the bending in-plane vibrations of the carbon–hydrogen bonds (βC–H), e.g., at 1517, 1484, 1446 cm^−1^ (in the spectrum of CA), and bending our-of-plane vibrations (γC–H) located at 804 cm^−1^ (in the spectrum of CA). There were also many bands assigned to the deforming in-plane vibrations of the aromatic ring (def_ring_) (at 765, 736, 715, 583 cm^−1^ in the spectrum of CA) as well as out-of-plane (def_ringou_) (located at 877, 659, 596 cm^−1^ in IR spectrum of CA). Characteristic bands assigned to the vibrations of hydroxyl groups of caffeic acid moiety βOH were observed at 1167, 1120 cm^−1^ in the IR spectrum of CA. These bands were shifted toward lower wavenumbers in the IR spectra of sodium salt and complexes. Bands assigned to the bending out-of-plane vibrations γOH at 565, 504 cm^−1^ in the IR spectrum of cichoric acid were shifted toward higher wavenumbers or disappeared in the spectra of complexes. Other characteristic bands from the spectra of the studied compounds were assigned to the oscillations of the carbonyl group from the carboxylic group of caffeic acid and the carbon-carbon bond of C=C of caffeic acid. They occurred in the IR spectrum of CA at 1682 and 1624 cm^−1^, respectively. In the complexes, the first band was shifted toward higher wavenumbers, whereas the second band disappeared in the spectra of the complexes.

### 3.2. Theoretical Study

**Figure 3:**
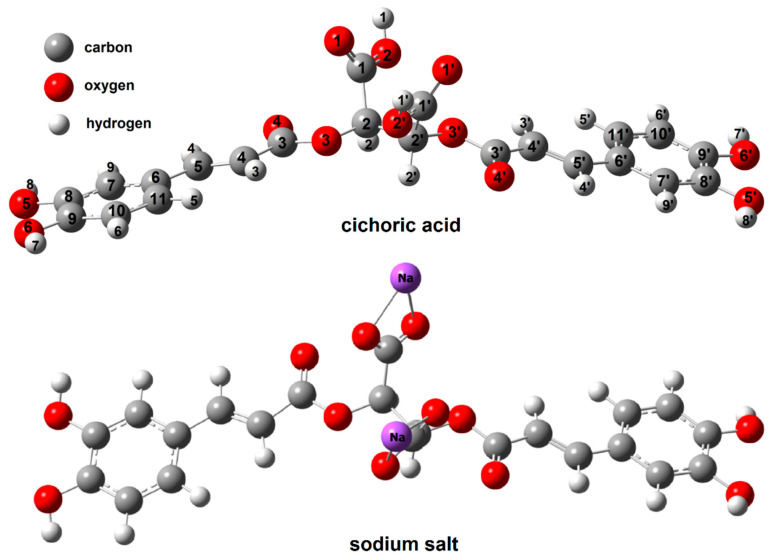
The structure of cichoric acid and sodium cichorate calculated in HF/6-311++G(d,p).

**Figure 4:**
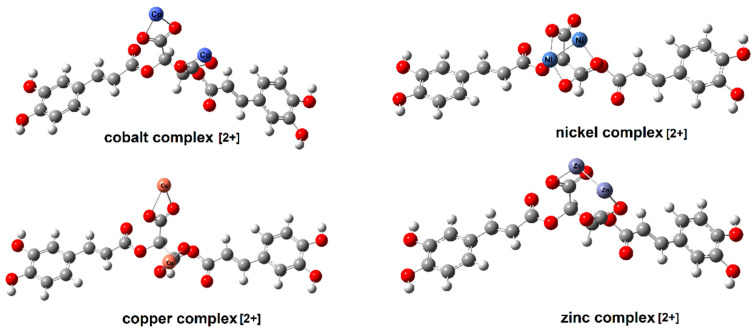
The structure of cation complexes (2+) cobalt, nickel, copper and zinc with cichoric acid calculated in HF/6-311++G(d,p).

Figure 3 shows the optimized structures of cichoric acid and sodium salt, Figure 4 shows and copper(II), zinc(II), cobalt(II) and nickel(II) cation (2+) complex. The calculated bond lengths were analysed and the geometrical aromaticity indexes were calculated to determine the effect of the metal cations on the aromaticity of a ligand.

The results of the calculations are presented in Table 2. The aromaticity of sodium salt and the Cu(II) complex was similar to that of cichoric acid. The differences between the values of the geometric indices of aromaticity calculated for ligand and metal compounds were small. A slight stabilization of the aromatic system was observed after ligand complexation by copper and sodium as a slight increase in the values of their aromaticity indices. In the case of complexes of cichoric acid with zinc, nickel and cobalt, a decrease in the value of their aromaticity indexes was observed, which indicates higher aromaticity of the ligand compared with theses complexes.

The carbon–oxygen bond lengths of the carboxylate groups in sodium and copper cichorates (C1–O1, C1–O2, C1′–O1′, C1′–O2′) were aligned. The C2–O3 and C1–C2 bonds were longer in complexes compared to ligand molecule. The C3–O3 bond in the carboxylic group in the caffeic acid moiety were slightly shortened in complexes (Cu, Na) compared to ligand (Table 3), while in Co, Ni and Zn complexes it has been extended.

**Figure 5:**
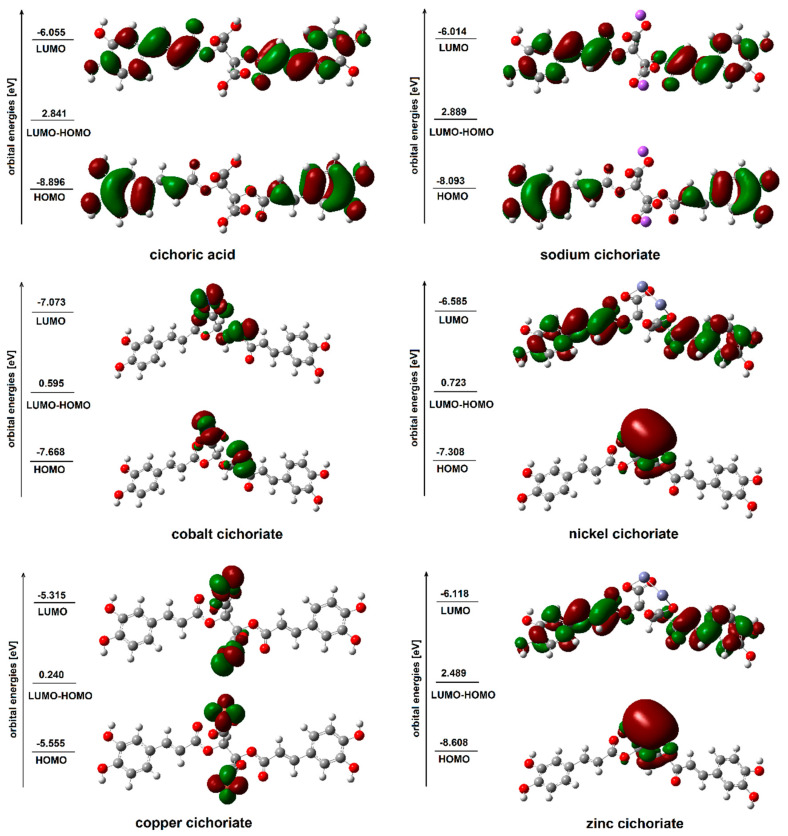
HOMO and LUMO molecular orbitals of cichoric acid and sodium, cobalt, nickel, copper and zinc cichorates (2+).

The values of the energy of HOMO and LUMO orbitals as well as the differences between these values for acid, sodium salt and copper(II), zinc(II), nickel(II) and cobalt(II) cation complexes were also calculated. The shape of the orbitals is shown in Figure 5. The energy of HOMO orbital was similar for the sodium salt and CA, whereas for the copper, zinc, cobalt and nickel complexes this value increased significantly.

The difference in the energy of HOMO and LUMO (ΔE) orbitals was greater for sodium salt, whereas ΔE for complexes were much smaller than for the acid. The values of the energy of HOMO and LUMO orbitals reflects the chemical reactivity of the compound with free radicals. A soft molecule—a molecule with a small value of ∆E—is more polarized and more reactive than a hard one, because it easily offers electrons to an acceptor [33]. According to Suksrichavalita et al. [34,35], molecules with higher HOMO energies have lower SOD activity (extinction of superoxide radicals carried out in the body by the enzyme dismutase) [36]. It was also found that the calculated energies HOMO and LUMO correlated well with the SOD activity, which was confirmed in experiment [37]. The calculated values of HOMO and LUMO energy as well as the differences between these energies for the studied systems allowed us to conclude that the Cu, Zn, Ni, and Co complexes of cichoric acid may show higher activity in scavenging free radicals than acid.

### 3.3. UV Study

**Figure 6:**
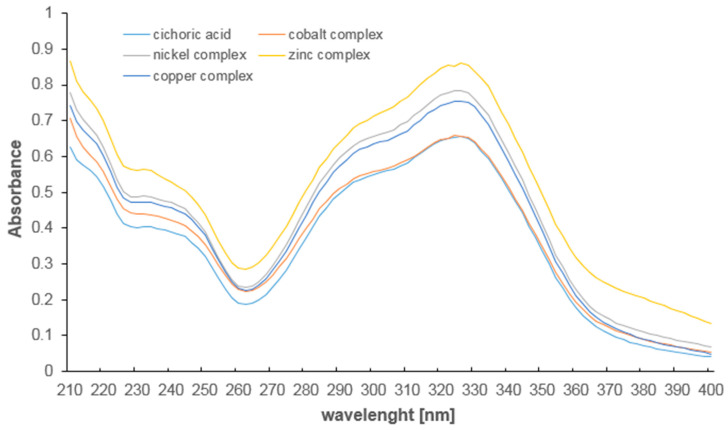
The absorption spectra of cichoric acid and complexes with copper, zinc, cobalt, nickel (210–400 nm, aqua solution 5 × 10^−5^ mol/dm^3^).

In the UV spectra of studied compounds two maximum of absorption occurred which correspond to the π→π* transitions in the aromatic ring of caffeic acid moiety (Figure 6, Table 4). In the UV spectra of acid the maximum occurred at λ_max1_ = 327.5 nm and λ_max2_ = 233.0 nm. In the spectra of complexes these band were hipsochromically shifted. This indicates a slight influence of metal ions on the electronic charge distribution in the ring of the molecule. The presence of copper and zinc ions caused weaker effect than nickel and cobalt.

### 3.4. Antioxidant Study

#### 3.4.1. Ferric-Reducing Antioxidant Power (FRAP), Cupric-Reducing Antioxidant Capacity (CUPRAC), DPPH Assays

**Figure 7:**
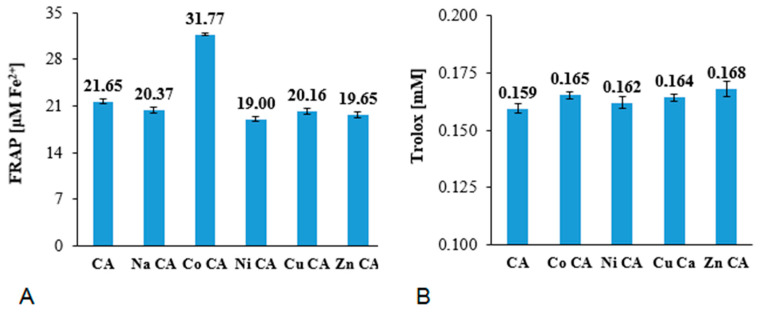
Antioxidant activity measured in ferric-reducing antioxidant power (FRAP) (**A**) and cupric-reducing antioxidant capacity (CUPRAC) (**B**) assays of cichoric acid (CA) and its metal compounds at the concentrations of 6 μM.

The FRAP values obtained for the studied compounds at the concentration of 6 μM are shown in Figure 7A. The ferric-reducing antioxidant activity of CA and its metal complex did not differ much. The results pointed that cobalt(II) complex with cichoric acid possessed the strongest antioxidant activity measured by the FRAP assay (Co CA: 31.77 μM Fe^2+^), whereas other studied metal compounds showed slightly lower ferric-reducing activity than cichoric acid. The CUPRAC assay (Figure 7B) revealed slightly higher reducing activity of Cu(II), Zn(II), Ni(II) and Co(II) complexes of cichoric acid than ligand alone.

**Figure 8:**
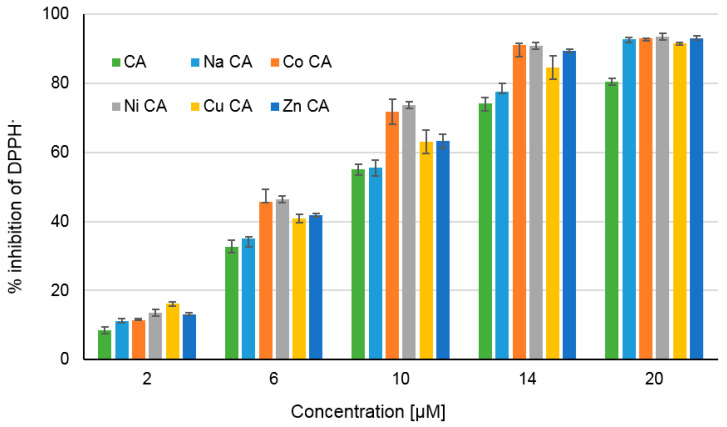
The percentage of DPPH inhibition by cichoric acid (CA) and its metal compounds in the range of their concentration 2–20 µM.

The antiradical activity of tested substances is depicted in Figure 8 as a percentage of DPPH radical inhibition. The degree of inhibition of DPPH by CA and its metal compounds depends on the concentration of tested compounds. With the increase in their concentration (from 2 to 20 μM) the inhibition% increases as well in the range of 8–93%. Moreover sodium salt of CA as well as Co(II), Ni(II), Cu(II) and Zn(II) CAs possess higher antiradical activity than the ligand. In each tested concentrations cobalt and nickel cations caused the highest increase in the antiradical activity of ligand. The calculated EC_50_ parameters for tested compounds raised in the order: Ni CA (EC_50_ = 7.10 µM) < Co CA (EC_50_ = 7.24 µM) < Zn CA (EC_50_ = 7.70 µM) < Cu CA (EC_50_ = 7.80 µM) < Na CA (EC_50_ = 8.95 µM) < CA (EC_50_ = 9.36 µM). The lower value of the EC_50_, the higher antiradical activity of the compound.

On the basis of the parameters obtained in the performed herein antioxidant tests for CA and reported previously for strong antioxidant L-ascorbic acid [38] it can be clearly stated that cichoric acid has a high antioxidant potential. In the experiment (conducted in the same conditions as here) the EC_50_ for *L*-ascorbic acid in DPPH tests was: 10.87 μM. The complexation of cichoric acid by transition metal ions changes the structure of the tartaric acid moiety where the metal is coordinated. The changes in bond lengths and angles within caffeic acid moiety were smaller. The calculated aromaticity indices for the studied complexes showed that metal ions such as nickel, cobalt and zinc have a greater effect on the aromatic system of the acid than copper. The changes in the molecular structure of CA ligand due to metal complexation cause differences in the biological activity of complexes. Already the literature data showed that complexation of cichoric acid with 3d-transition metals affected the microbiological and cytotoxic properties of the ligand [15]. FRAP, DPPH, CUPRAC tests and theoretical calculations have shown that complexation with metals such as Cu(II), Zn(II), Ni(II) and Co(II) changes the antioxidant properties of cichoric acid. In a DPPH test the complexes with Co(II) and Ni(II) mostly increased the antiradical activity of ligand. Smaller increased was observed in the case of Cu(II) and Zn(II) complexes. The FRAP assay revealed that Co(II) mostly increased the reducing properties of cichoric acid compared with other metals. The reducing activity of metal complexes in the CUPRAC test was higher than the ligand, but non-significant differences between particular complexes were observed. The reducing properties in the both tests are associated with the transfer of a single electron from the antioxidant in order to reduce iron or copper cations. Complexation of cichoric acid with 3d-transition metals facilitates electron transfer, which increases antioxidant activity of complexes compared to ligand.

Cichoric acid can also affect the body’s absorption of iron ions. In a well-balanced diet, more than 2/3 of the total amount of iron is a poorly soluble non-haem iron Fe^3+^. In duodenal enterocytes, absorption of non-haem iron Fe^3+^ depends on the activity of the bivalent metal transmembrane transporter (DMT1, divalent metal transporter 1), which, however, does not accept Fe^3+^ iron as a substrate. Therefore, it is required to reduce food iron before its intestinal uptake. This reaction is catalyzed by duodenal cytochrome b (Cybrd1)—ferroreductase—present on the surface of mature intestinal absorption cells [39]. Cichoric acid, like other phenolic acids, which is confirmed by FRAP tests, has the ability to reduce Fe^3+^ to the Fe^2+^, which is absorbed better in the digestive system [40]. The presence of other metal ions does not significantly reduce the ability of cichoric acid to reduce iron, which is confirmed by research. So it can be expected that cichoric acid consumed in the diet can have a positive effect on the absorption of iron. Moreover the effective concentration of CA and its metal compounds in scavenging DPPH radical is even lower than that obtained for *L*-ascorbic acid. This creates the possibility of application of CA and its metal complexes in bio-formulations or biopreparations as strong antioxidants. The metal compounds of CA may be of special interest because they possess different physico-chemical properties than the ligand alone (e.g., increased solubility in the case of sodium salt), better lipophilicity [38] and they are an additional source of macro- and microelements.

#### 3.4.2. SOD (Superoxide Dismutase Activity Assay)

**Figure 9:**
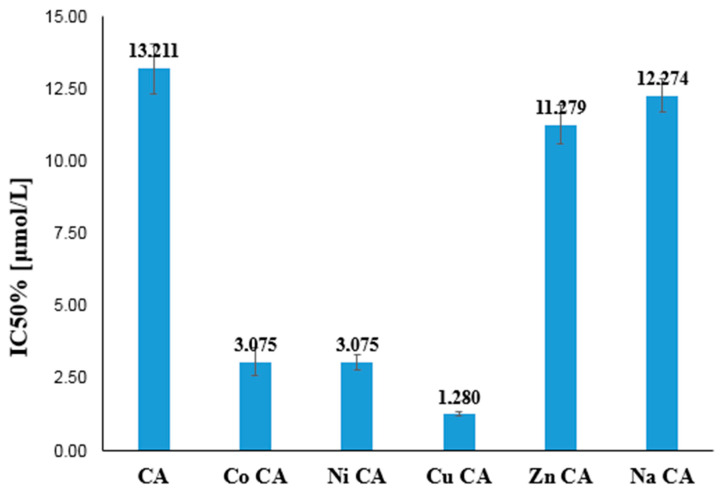
Antioxidant activity (measured using O_2_ radical assay) of cichoric acid (CA) and its metal complexes.

Metal complexes of cichoric acid showed better antiradical activity of against superoxide radical than cichoric acid (Figure 9). The Cu(II) complex with cichoric acid possessed the highest SOD-mimic activity among studied compounds. Ni(II) and Co(II) also strongly increased the antioxidant activity of ligand (but not so much as Cu(II) complex). High antioxidant activity of Co(II) and Ni(II) complexes was shown as well in DPPH and FRAP tests; whereas the coordination with Zn(II) ion to a small extent increased the activity of the ligand.

Some of the transition metal complexes, including copper, may react with a superoxide anion radical in a way resembling activity of native superoxide dismutases. Hence, they were called SOD mimetics (SODm) [41]. In the case of ligands such as bipyridyl and phenanthroline, the presence of a copper(II) cation in their coordination sphere caused an increase in the reactivity of the complex with a superoxide anion radical compared to ligand alone. On the other hand, a decrease in the antioxidant activity of these compounds occurred when dipeptides were included in the copper(II) complex [42]. The heterocyclic nitrogen base ligands have a significant impact on the increase in the reactivity of copper(II) complexes with superoxide anion radicals. Although copper(II) complexes show a lower antioxidant activity than the SOD enzyme, they are important as potential superoxide dismutase mimetics due to their low molecular weight [43].

Antioxidant properties of cichoric acid complexes were also tested in the superoxide radical (SOD) test. This showed that the zinc complex has the antioxidant capacity slightly higher than cichoric acid. The cobalt and nickel complexes exhibited a much higher antioxidant capacity than cichoric acid, and the highest superoxide radical removal capacity among the studied compounds showed the copper complex.

Antioxidative activity can be also discussed on the basis of the theoretical parameters calculated by quantum-chemical methods, e.g., the energy of HOMO and LUMO orbitals. According to Schepetkin et al. the calculated HOMO and LUMO energies correlated well with SOD activity, which was represented as log (1/IC_50_) (experimental results) [37]. Electron affinity (EA) is a good descriptor to characterize the scavenging activity of superoxide radicals [36]. Compounds with the lowest EA value have the highest electron transfer capacity, thus giving the highest SOD activity [36]. The theoretical calculations presented showed that the copper(II) complex with cichoric acid had the lowest EA value. This confirms the highest ability to remove superoxide radical by copper(II) complex determined experimentally in the SOD test. The high antioxidant activity of the Cu(II) complex with cichoric acid can be explained by the mechanism of reaction of the complex with radicals. The scavenging reaction with superoxide radical engages: (i) the four hydroxyl groups of cichoric acid (hydroxyl catechol group from the caffeic acid moiety) and (ii) Cu(II), which is reduced to Cu(I) supporting the anti-radical process. Curcumin-copper complexes act similarly and in the reaction with a superoxide radical behave like SOD-mimic superoxide dismutase [44].

## 4. Conclusions

The spectroscopic studies of cichoric acid and the selected complexes of cichoric acid obtained in solid state and solution allowed the structures of the studied compounds and their composition to be determined. The theoretical calculations performed for the acid and selected complexes (cations 2+) allowed proper assignment of the vibrational bands from the IR spectra. The metal cations are bound by the carboxylic groups of the tartaric acid moiety (the structural part of cichoric acid). In aqueous solutions (pH stabilized with a buffer of pH = 7.4) the ratio of ligand:metal is 1:2. The general formula of studied compounds is [C_22_O_12_H_16_Na_2_]·2H_2_O and [C_22_O_12_H_16_M_2_Cl_2_]·4H_2_O for M = Ni(II), Zn(II) and Co(II) complexes and [C_22_O_12_H_16_M_2_Cl_2_]·2H_2_O for the copper complex. The calculations of the energy of the HOMO and LUMO orbitals allowed the prediction of the anitoxidant activity of the tested compounds.

The antioxidant activity of cichoric acid and its metal compounds was established by the use of DPPH, FRAP, CUPRAC and SOD-mimic assays. Cichoric acid has high antioxidant activity, but the complexation with metal cations may increase its antioxidant properties. The DPPH and FRAP assays showed that cobalt(II) complex of cichoric acid possessed higher antioxidant activity than other complexes and ligands alone. Moreover the DPPH and CUPRAC assays indicated that all metal cations enhanced the antioxidant properties of ligand, what was not confirmed by the use of FRAP assay. The Cu(II) complex of cichoric acid showed the highest activity against superoxide radical among studied complexes. It revealed that the Cu(II) complex behaves like SOD mimetics. Moreover the results of the theoretical calculations of the energy of HOMO and LUMO orbitals indicated that the copper(II) complex had higher activity than cichoric acid. Although all data obtained indicate the higher antioxidant activity of metal complexes of CA than the CA alone, there is no clear dependency between the type of metal and its effect on the antioxidant properties of CA. This can be explained by different type of reaction taking place during particular tests which engage electrons (SET; single electron transfer; in the FRAP and CUPRAC methods) or hydrogen and/or electron transfer from antioxidant (different mechanisms in the DPPH assay: (a) HAT; hydrogen atom transfer, (b) PCET; proton-coupled electron transfer), (c) SPLET; sequential proton loss electron transfer and ET-PT, electron transfer proton loss). Moreover the different types of solvents applied in the antioxidant assay (water or methanol) affect the strength of hydrogen bonding between molecules and solvents, and as a consequence the electron and/or hydrogen donating properties of antioxidants. Because the type of metal cation strongly affects the type of metal coordination by CA and the electronic charge distribution in the ligand molecule, it is supposed that the type of metal cation influences the antioxidant properties of the ligand as well. Therefore it is not possible to synthetize one strong universal antioxidant exhibiting the same strong antiradical properties in relation to all radicals or reducing properties toward Cu(II) or Fe(III) ions.

Polyphenols are a very large group of compounds with a broad spectrum of biological activity. Their therapeutic effect results from their proven antioxidant and anti-tumor activity. Some of these compounds are at the stage of clinical trials, but so far none of them is available as an anti-cancer drug, mainly due to their poor pharmacokinetic profile. However, their ability to complex metals resulted in the possibility of improving their pharmacological properties. Although in vitro studies show a significant improvement in the antioxidant, antiproliferative and proapoptotic properties of these compounds after metal chelation, further studies are necessary to confirm this phenomenon. Structural modifications of polyphenols also give some hope for improving the bioavailability of these compounds and improving their absorption in the digestive system, which is generally at a very low level.

## Figures and Tables

**Table 1:** Wavenumbers (cm^−1^), intensities and assignments of bands occurring in the IR (KBr, density functional theory (DFT) and Hartree–Fock (HF)) and Raman spectra of cichoric acid, sodium salt and 3d metal complexes.

Cichoric Acid						Sodium Salt				Complexes *						Assignments
										Copper			Nickel	Zinc	Cobalt	
Experimental		Theoretical				Experimental		Theoretical		Exp	Theoret		Exp	Exp	Exp	
IR	Raman	DFT	Int	HF	Int	IR	Raman	HF	Int	IR	HF	Int	IR	IR	IR	
3412 s		3758	109.22	3741	138.74	3423		3742	85.89	3454 vs	3742	3.66	3441 s	3452 vs	3406	νOH_caff_
1746 m		1798	236.02	1809	331.95											ν_as_COOH_tart_
1716 s		1788	352.52	1797	512.19											ν_as_COOH_tart_
1682 vs	1681 s	1732	6.19	1744	18.96	1699 s	1703 w	1735	7.47	1721 s	1736	9.23	1698 s	1692 m		νC = O_caff_, νC = C_alcaff_
1624 s	1627 vs	1730	495.5	1742	697.97			1732	686.64		1731	669.21				νC = O_caff_, νC = C_alcaff_
						1627 vs		1580	1255.35	1625 s	1570	1105.71	1630 s	1629 vs	1626 s	ν_as_COO^−^
								1578	218.51		1568	184.99			1605 s	ν_as_COO^−^
1606 vs	1609 vs	1644	474.78	1640	680.3	1599 vs	1603 vs	1650	489.32		1649	529.87				νCC_ar_, νC = C_alcaff_
1517 s	1516 m	1605	901.82	1601	487.27	1522 s	1521 vw	1607	181.58	1522 m	1606	220.16	1522 m	1524 m	1521 m	νCC_ar_, νC = C_alcaff_, βC-H_ar_, βC-H_alcaff_,
1484 w	1482 m	1600	106.27	1593	122.69			1594	127.39		1594	132.35				βC-C_ar_, βC-H_ar_, νCC_ar_,
1446 w		1528	266.54	1515	417.92	1449 m	1456 vw	1515	431.85	1446 m	1514	428.92	1446 m	1449 m	1445 m	βC-H_ar_
						1385 s	1381 vw	1447	204.62	1385 m	1446	185.54	1385 m	1384 s	1385 m	ν_s_COO^−^
								1430	139.79		1430	129.3				ν_s_COO^−^
		1437	94.71	1415	171.64			1414	120.46		1414	122.76				νCC_ar_, βC-H_ar_,
1362 s	1364 m	1393	42.79	1412	41.26											βC-H_altart_
1337 m	1342 vw	1386	1.94	1405	0.01											βC-H_altart_
1300 s	1303 m	1354	15.64	1327	3.29		1304 vw	1324	343.53	1258 s	1325	329.41		1304 m		def_ring_, νCC_ar_, βC-H_ar_, βC-H_altart_
1280 m	1272 w	1335	9.92	1312	95.46		1287 vw	1312	54.87		1313	32.33	1285 m	1285 s		βC-H_ar_, βC-H_altart_
1246 vs	1249 m			1302	19.17	1259 vs	1262 vw	1303	140.18		1305	193.71	1260 m	1259 s	1260 s	βC-H_altart_
1218 vs	1213 w	1306	216.72	1288	116.76		1203 vw	1286	67.15		1286	69.23				def_ring_, βC-H_ar_, βC-H_alcaff_
1167 s	1170 w	1185	106.51	1164	72.74	1153 vs	1161 vw						1154 m	1154 s	1156 s	βC-H_ar_, βC-H_alcaff_
1120 s	1125 w	1169	35.07	1161	1263.96	1117 s	1120 vw			1117 m			1116 m	1117 s	1115 s	βC-H_ar_, βO-H_caff_
1078 m	1037 w	1166	473.39	1153	483.48	1048 m	1049 vw	1153	8.67	1052 m	1153	9.64	1049 w	1051 m	1051 m	βC-H_tart,_βC-C_tart_
990 m	992 vw	1067	215.16	1072	8.57			1066	168.31		1066	391.83				νC(2)-O(3), νC(2′)-O(3′),
976 s	976 vw	1055	10.8	1066	338.46	977 m	979 vw	1065	236.09	981 w	1065	4.89	979 w	979 m	976 m	δC(2)-C(2′)
		976	1.85	965	0.25			967	0.01		966	0.01				βC = C_alcaff_
		961	41.67	963	18.6			965	43.22		964	42.67				def_ring_, βC = C_alcaff_
						930 vw	903 vw	956	24.73	924 w	956	25.84	930 vw	930 w	935 w	γ_s_COO^−^
		950	29.56	945	9.87			937	5.92		937	5.85				def_ring_
898 w	896 vw	940	1.08	936	5.25		880 vw	929	19.11		928	19.28		896 vw		τC(2)-C(2′)
877 w	868 vw							896	18.1		895	19.28				def_ring ou_
867 w	862 vw	870	12.29	885	17.44											γC-H_ar_, γC-H_alcaff_
						853 w	860 vw	870	18.44	868 w	871	10.97	851 w	853 w	851 m	β_s_COO^−^
								869	8.1		869	20.35				β_s_COO^−^
804 m	810 vw	835	66.84	862	96.44	810 m	812 vw	863	86.38		863	88.76	810 w	812 m	809 m	γC-H_ar,_ γC-H_alcaff_
		826	52.13	826	15.3			813	80.19		813	82.51				τC(2)-C(2′)
		813	0.34	812	72.65			813	2.37		813	0.22				δC(2)-H, δC(2′)-H
		791	47.47	782	5.37			795	44.83		794	48.66				γC-H_ar_
765 w	765 vw	787	41.02	779	66.92	782 w	787 vw							779 w	782 m	def_ring_, βC(1)-C(2), βC(1′)-C(2′)
736 w	730 vw	772	49.56	762	12.51			763	14.81		763	13.91		729 w		def_ring_, νC-C_ar_
715 vw		731	5.87	740	5.32	715 w	719 vw							725 w		def_ring_, γCOO, γC-H_alcaff_
698 vw	699 vw	723	5.23	739	2.12			746	3.41		745	4.05	698 w	695 m		γCOOH
680 w	685 vw	703	1.14	727	4.36	688 w		727	1.11		726	2.16	691 m		685 m	def_ring_, γCOOH
659 w	664 vw	692	0.32	712	11.57		659 vw	704	3.38		704	3.24	676 w			def_ring ou_
A596 m	597 vw	587	30.45	574	113.89	596 w		573	16.53	591 m	586	4.11	598 m	598 m	595 m	def_ring ou_
583 m	584 vw	579	46.79	566	118.25			562	1.28		562	1.42				def_ring_
576 m	576 vw	569	79.93	553	170.63			560	34.96		559	37.81			566 m	def_ring_
565 w	565 vw	563	24.82	549	19.81		546 vw	550	6.3	550 m	550	7.58	574 w	568 m		γO-H_caff_
						521 w		539	40.51	521 m	536	39.39	520 m	520 m	521 m	β_as_COO^−^
								472	39.33		471	46.44				β_as_COO^−^
504 w	504 vw	542	0.55	517	7.63											γO-H_caff_

ν—stretching vibrations, β—bending in-plane, γ—bending out-of-plane, δ—rotating, τ—swaying, def_ring_—deformation of the ring in-plane, def_ringou_—deformation of the ring out-of-plane, vs—very strong, s-strong, m—medium, w—weak, vw—very weak, s—symmetric oscillations, as—asymmetric oscillations, caff—caffeic acid, tart—tartaric acid, ar—aromatic, al.—aliphatic, complexes * are cations (2+).

**Table 2:** Geometrical parameters calculated in HF/6-311++G(p, d).

	Cichoric Acid	Sodium Cichorate *	Copper(II) Cichorate (2+) **	Zinc (II) Cichorate (2+)	Nickel(II) Cichorate (2+)	Cobalt (II) Cichorate (2+)
Energy (Hartree)	−1742.43	−2065.08	−5019.01	−5296.64	−4766.91	−4503.67
Dipole moment (D)	1.27	11.19	8.88	2.21	3.83	9.02
Aj	0.995	0.996	0.996	0.992	0.989	0.995
BAC	0.896	0.907	0.911	0.876	0.861	0.903
HOMA	0.988	0.990	0.990	0.944	0.936	0.898
EN	0.001	0.001	0.008	0.338	0.041	0.001
GEO	0.011	0.009	0.001	0.018	0.041	0.010
I6	94.48	95.04	95.23	92.90	92.44	94.83
HOMO (Hartree)	−0.32693	−0.32718	−0.20416	−0.31634	−0.26857	−0.2818
LUMO (Hartree)	−0.22251	−0.22100	−0.19533	−0.22485	−0.24200	−0.25994
HOMO (eV)	−8.896	−8.903	−5.555	−8.608	−7.308	−7.668
LUMO (eV)	−6.055	−6.014	−5.315	−6.118	−6.585	−7.073
Energy gap	2.841	2.889	0.240	2.489	0.723	0.595
Ionization potential, I = −E_HOMO_	8.896	8.903	5.555	8.608	7.308	7.668
Electron Affinity, A = −E_LUMO_	6.055	6.014	5.315	6.118	6.585	7.073
Electronegativity, χ = (I + A)/2	7.475	7.455	5.440	7.363	6.947	7.371
Chemical potential, μ = −(I + A)/2	−7.475	−7.455	−5.440	−7.363	−6.947	−7.371
Chemical hardness, η = (I − A)/2	1.425	1.445	0.120	1.245	0.362	0.297
Chemical softness, S = 1/2η	0.351	0.346	4.167	0.402	1.383	1.681
Electrophilicity index, ω = μ^2^/2η	−2.623	−2.580	−22.667	21.778	66.743	91.332

* Neutral molecule, ** cation (2+).

**Table 3:** Bond lengths for cichoric acid, sodium salt and cation (2+) complex of Cu(II), Co(II), Ni(II), Zn(II).

	Cichoric Acid		Sodium Salt	Copper(II) Complex Cation	Cobalt(II) Complex Cation	Nickel(II) Complex Cation	Zinc (II) Complex Cation
	B3LYP/6-311++G(d,p)	HF/6-311++G(d,p)	HF/6-311++G(d,p)	HF/6-311++G(d,p)	HF/6-311++G(d,p)	HF/6-311++G(d,p)	HF/6-311++G(d,p)
C1–O1	1.201	1.178	1.239	1.239	1.233	1.309	1.268
C1–O2	1.344	1.317	1.236	1.236	1.247	1.309	1.275
C1–C2	1.536	1.528	1.535	1.535	1.521	1.568	1.533
O3–C2	1.422	1.398	1.410	1.410	1.404	1.416	1.427
C3–O3	1.371	1.336	1.319	1.319	1.335	1.376	1.370
C3–O4	1.210	1.186	1.188	1.188	1.184	1.213	1.216
C3–C4	1.466	1.473	1.484	1.483	1.479	1.466	1.468
C4–C5	1.345	1.327	1.325	1.325	1.326	1.348	1.348
C5–C6	1.457	1.470	1.474	1.474	1.472	1.457	1.457
C6–C11	1.402	1.386	1.386	1.386	1.386	1.404	1.404
C10–C11	1.388	1.384	1.384	1.384	1.384	1.391	1.391
C9–C10	1.396	1.382	1.382	1.382	1.382	1.397	1.397
C8–C9	1.406	1.394	1.393	1.393	1.393	1.410	1.410
C8–C7	1.388	1.378	1.379	1.379	1.378	1.389	1.389
C7–C6	1.407	1.396	1.395	1.395	1.396	1.410	1.410
C8–O5	1.364	1.348	1.349	1.349	1.349	1.365	1.365
C9–O6	1.360	1.344	1.347	1.347	1.346	1.361	1.361
C1′–O1′	1.201	1.178	1.239	1.239	1.233	1.214	1.268
C1′–O2′	1.344	1.317	1.236	1.236	1.247	1.377	1.275
C1′–C2′	1.536	1.528	1.535	1.535	1.521	1.568	1.533
O3′–C2′	1.422	1.398	1.410	1.410	1.404	1.456	1.427
C3′–O3′	1.371	1.336	1.319	1.319	1.335	1.400	1.370
C3′–O4′	1.210	1.186	1.188	1.188	1.184	1.209	1.216
C3′–C4′	1.466	1.473	1.483	1.483	1.479	1.461	1.468
C4′–C5′	1.345	1.327	1.325	1.325	1.326	1.352	1.348
C5′–C6′	1.457	1.470	1.474	1.474	1.472	1.453	1.457
C6′–C11′	1.402	1.386	1.386	1.386	1.386	1.405	1.404
C10′–C11′	1.388	1.384	1.384	1.384	1.384	1.390	1.391
C9′–C10′	1.396	1.382	1.382	1.382	1.382	1.398	1.397
C8′–C9′	1.406	1.394	1.393	1.393	1.393	1.411	1.410
C8′–C7′	1.388	1.378	1.379	1.379	1.378	1.388	1.389
C7′–C6′	1.407	1.396	1.395	1.395	1.396	1.412	1.410
C8′–O5′’	1.364	1.348	1.349	1.349	1.349	1.362	1.365
C9′–O6′	1.360	1.344	1.347	1.347	1.346	1.358	1.361

**Table 4:** The absorbance maximum in the UV spectra of cichoric acid and its metal complexes.

	Cichoric Acid	Cobalt Cichorate	Nickel Cichorate	Copper Cichorate	Zinc Cichorate
λ_max1_ [nm]	327.5	324.0	325.0	325.0	326.0
λ_max2_ [nm]	233.0	230.0	232.0	232.0	232.0
